# Metronomic temozolomide as second line treatment for metastatic poorly differentiated pancreatic neuroendocrine carcinoma

**DOI:** 10.1186/s12967-016-0857-1

**Published:** 2016-05-03

**Authors:** C. De Divitiis, C. von Arx, A. M. Grimaldi, D. Cicala, F. Tatangelo, A. Arcella, G. M. Romano, E. Simeone, R. V. Iaffaioli, P. A. Ascierto, S. Tafuto, On behalf of the European Neuroendocrine Tumor Society (ENETS) Center of Excellence-Multidisciplinary Group for Neuroendocrine Tumors in Naples (Italy)

**Affiliations:** Department of Abdominal Oncology, Istituto Nazionale Tumori, IRCCS–Fondazione “G. Pascale”, Naples, Italy; Department of Clinical Medicine and Surgery, University of Naples Federico II, Naples, Italy; Melanoma, Cancer Immunotherapy, and Innovative Therapy Unit, Istituto Nazionale Tumori, IRCCS–Fondazione “G. Pascale”, Naples, Italy; Unit of Interventional Neuroradiology, Department of Advanced Biomedical Sciences, “Federico II” University, Naples, Italy; Department of Diagnostic Pathology and Laboratory, Istituto Nazionale Tumori, IRCCS–Fondazione “G. Pascale”, Naples, Italy; IRCCS Neuromed, Località Camerelle, Pozzilli–Isernia, Italy

**Keywords:** Pancreatic neuroendocrine carcinoma, Temozolomide, Second line therapy, Metronomic treatment, Neuroendocrine tumors, Immunotherapy

## Abstract

Neuroendocrine Neoplasms (NEN) are a group of heterogeneous malignancies derived from neuroendocrine cell compartment, with different roles in both endocrine and nervous system. Most NETs have gastroentero-pancreatic (GEP) origin, arising in the foregut, midgut, or hindgut. The 2010 WHO classification divides GEP-NETs into two main subgroups, neuroendocrine tumors (NET) and neuroendocrine carcinomas (NEC), according with Ki-67 levels. NET are tumors with low (<20 %) Ki-67 value, and NECs, including small cell lung carcinomas and Merkel Cell carcinomas, are all NETs with high Ki-67 levels (>20 %–G3). Poorly differentiated neuroendocrine carcinomas (NEC) are usually treated with cisplatin-based chemotherapy regimens. Here we present a case of a patient with pancreatic NEC progressing after cisplatin and etoposide, treated with temozolomide as palliative, second line treatment. According with the poor Performance Status (PS = 2) and to reduce the toxicity of the treatment was chosen an intermittent dosing regimen of metronomic temozolomide (75 mg/m^2^/day—one-week-on/on-week-off). MGMT resulted methylated. On July 2014 the patient started the treatment. On August 2014 the patient obtained a significant clinical benefit (PS = 0) and the total body CT scan performed on October 2014 showed a RECIST partial response on all the sites of disease. No drug-related side effects were reported by the patient. After 18 months of therapy the treatment continues without significant toxicity, and with further remission of the metastases. Treatment with metronomic “one-week-on/on-week-off” Temozolomide can be considered a good treatment option in patients with poor performance status, affected by pNEC with MGMT methylation.

## Background

Neuroendocrine tumors (NETs) are a heterogeneous group of malignancies derived from neuroendocrine cell compartment [1, 2], with roles in both endocrine and nervous system. The treatment strategy of NET varies according with several factors, such as tumor differentiation, stage at diagnosis, and presence or absence of symptoms related to hormonal secretion. Surgical resection represents the traditional treatment of NETs and it is the only curative approach. However, the surgical excision is not always possible because most of the patients are metastatic at the diagnosis, with regional or distant metastases observed in about 50 % of patients. 65 % of pancreatic NETs (pNET) are diagnosed when the disease is already metastatic [3, 4]. There is a high unmet medical need to control tumor growth in patients with advanced (unresectable or metastatic) NET. In patients with advanced NETs, the treatment goal is to control hormone-related symptoms (if the tumor is functional), tumor growth, and prolong overall survival of the patients. Biotherapy with somatostatin analogs (SSAs) remains the mainstay of symptomatic therapy [5, 6]. More recently, the PROMID study has shown that octreotide LAR (long-acting release) increased time to tumor progression (TTP), as compared with placebo, from 6 to 14.3 months in treatment-naive patients with advanced NET of midgut origin [7]. These findings have been confirmed and extended by the recent CLARINET trial with lanreotide [8], published in 2014. This trial showed a significantly prolonged progression-free survival (PFS) in patients with metastatic grade 1 or 2 (Ki-67 <10 %) enteropancreatic neuroendocrine tumors. Somatostatin analogues can inhibit the tumor growth and stabilize disease irrespective of the hormonal activity of the tumor. Cytoreductive surgery and regional ablation are used with palliative intent in metastatic disease. Local cytoreductive/ablative therapies include hepatic (chemo) embolization, percutaneous ethanol injection, cryotherapy, radiofrequency ablation, and selective internal radiation with Yttrium-90 labeled microspheres [9]. Their impact on survival has to be proven in larger, controlled trials. Peptide radionuclide receptor therapy (PRRT) seems to be a promising treatment option but there are not data available from randomized controlled trials and this therapy is not available worldwide [10].

Chemotherapy was, for years, the only therapeutic option for the treatment of metastatic pNET, with very contradictory results. Considering that Neuroendocrine Carcinomas (NECs) have common embryological origin and similar histologic morphology of small cell lung cancer and Merkel Cell carcinomas, the combination of cisplatin plus etoposide is usually the favorite treatment schedule of poor differentiated neuroendocrine tumors. Although this platinum-based combination treatment had shown interesting results in terms of response rate on undifferentiated NETs [11], there was a minimal impact on overall survival, so these results remain controversial. Actually the schedule cisplatin plus etoposide is only a virtual standard therapy. The traditional use of this scheme derives from old studies, with little statistical evidences due to the small number of patients enrolled in clinical trials. Therefore it is uncertain that cisplatin and etoposide can be considered the gold standard for the treatment of these tumors. Furthermore other drugs, as gemcitabine, oxaliplatin or temozolomide can be evaluated in the treatment of NEC.

Temozolomide (TMZ) is an imidazotetrazine derivative of the alkylating agent dacarbazine, which shows good central nervous system distribution. The use of TMZ is particularly indicated in the treatments of brain tumors, primary central nervous system lymphoma, neuroendocrine and pituitary tumors. TMZ was approved by the Food and Drug Administration (FDA) and European Medicines Agency (EMA) in 1999 for the treatment of multiform glioblastoma and anaplastic astrocytoma in case of recurrence or progression after standard therapy and in 2005 for newly diagnosed multiform glioblastoma in combination with radiotherapy and then as maintenance treatment.

The activity of TMZ in patients with metastatic neuroendocrine tumors has been evaluated in several trials [11, 12] which showed an interesting activity in terms of ORR, ranging from 25 to 70 %. [13–21]. TMZ showed a good activity in patients with NETs both in monotherapy both in association with other anti-cancer drugs as capecitabine, bevacizumab or thalidomide.

The association of TMZ plus capecitabine showed encouraging results. In vitro data indicate that this combination has a synergistic effect, inducing apoptosis in neuroendocrine tumor cell lines. A retrospective study of 17 patients with pNETs treated with TMZ plus capecitabine showed 1 complete response (6 %) and 9 partial responses (54 %), with a median duration of response of 284 days. All of the patients progressed during first-line treatment with escalating doses of sandostatin LAR, and 11 patients during multiagent chemotherapy (range 1–5 regimens) [22].

Moreover the association of TMZ plus capecitabine resulted particularly active in patients both with well, both with poor differentiated pancreatic neuroendocrine tumours.

In a trial reported by Strosberg et al. in 2011 [23], 30 patients with progressive metastatic pNETs, all chemotherapy-naïve, were treated with capecitabine (750 mg/m2 b.i.d., d. 1–14) plus temozolomide (200 mg/m^2^/day, d. 10–14) every 28 days. 70 % of the patients achieved a RECIST objective response, median progression-free survival was 18 months, and the 2 years survival rate resulted 92 %. Four patients (12 %) experienced grade 3 or 4 adverse events (Table 1).

**Table 1:** Main trial testing temozolomide in neuro-endocrine carcinomas (ORR;PFS;OS)

**References**	Regimens	ORR (%)	PFS (ms)	OS (ms)
Moertel [11]	Etoposide130 mg/mq iv ds 1–3 plus Cisplatin 45 mg/m^2^ iv ds 2–3	67	8	19
Ekeblad [14]	Temozolomide 200 mg/m^2^ os ds 1–5, q28	14	7	16
Welin [18]	Temozolomide 150–200 mg/m^2^ os ds 1–5, q28 ± Capecitabine 1000 mg os bid or 750 mg bid, ds 1–14	33	6	22
Strosberg [17]	Capecitabine 750 mg/m^2^ os bid, ds 1–14 plus Temozolomide 200 mg/m2 os ds 10–14, q28	70	18	n.d.

Therefore, these combination have a promising activity that should be evaluated in further studies with larger cohorts of patients to confirm the efficacy of these and to find the optimal schedule of association with other drugs. An interesting clinical trial from ECOG (ACRIN Cancer Research Group-E 2211) on these issues is ongoing.

Traditionally, neuroendocrine tumors have been classified by their anatomic site of origin. NETs can arise in many different areas of the body, and are most often located in the intestine, pancreas or the lungs. The various kinds of cells that can give rise to NETs are present in endocrine glands and are also diffusely distributed throughout the body. But all the NETs have a common embryologic origin from the neural crest. So between the neuroendocrine cancers are included different tumors as small cell lung cancer and Merkel cell carcinoma.

In the landscape of anti- tumor therapy, recently the immunotherapy has found a new field of application. Indeed, It has been proven that the tumors may adopt normal physiologic checkpoints for immunomodulation leading to an imbalance between tumor growth and host surveillance. Antibodies targeting the PD-1/PD-L1 checkpoint have shown dynamic and durable tumor regressions, suggesting a rebalancing of the host–tumor interaction. Nivolumab and Pembrolizumab are the anti-PD-1 antibodies that are currently the furthest in clinical development, and anti-PD-L1 agents under investigation include MPDL3280A, MEDI4736, and BMS-936559. These agents have been used to treat advanced melanoma, non-small cell lung cancer, renal cell carcinoma, bladder cancer and Hodgkin lymphoma, amongst other tumor types.

In the treatment of small cell lung cancer (SCLC), an aggressive neoplasm thought to be arising from lung neuroendocrine cells, several trial are ongoing to investigate PD-L1 and PD-1 expression patterns and the role of anti-tumour immunotherapy such as blockade of co-inhibitory immune pathways PD-1/PD-L1.

Ott and others [24] have observed, in the ongoing trial Keynote-028, that, in the treatment of patients with PD-L1 + SCLC who have progressed on prior platinum-based therapy, Pembrolizumab is generally well tolerated and, therefore, has promising antitumor activity. In fact, it has been demonstrated that out of the 135 patients with SCLC screened, 37 (27 %) had PD-L1 + tumors and of 16 treated with pembrolizumab (Pembrolizumab 10 mg/kg every 2 weeks for up to 2 years or until confirmed progression or unacceptable toxicity), 4/16 (25 %) evaluable patients had a partial response.

Pembrolizumab has promising antitumor activity also in the treatment of Merkel cell carcinoma (MCC). MMC is an aggressive neuroendocrine carcinoma of the skin, which can be distinguished from other malignancies by its expression of cytokeratin 20. Meantime, it is already known that, in cancer immunotherapy, dendritic cells (DCs) play a fundamental role in the dialog between innate and adaptive immune response, but several immunosuppressive mechanisms remain to be overcome. For example, a high number of CD4 + CD25 ++ Foxp3+ regulatory T-cells (Foxp3 + Tregs) have been observed in the peripheral blood and tumor microenvironment of cancer patients. On the basis of this, Ridolfi and others [12] conducted a study on DC-based vaccination in advanced melanoma, adding low-dose temozolomide to obtain lymphodepletion. They founded that the combined immunological therapy, at least as far as the DCR subgroup is concerned, effectively reduced the number of Foxp3 + Treg cells, which exerted a blunting effect on the growth stimulating effect of IL-2. However, this regimen, with its current modality, would not seem to be capable of improving clinical outcome.

Here we present a case of a patient with poor Performance Status (PS), affected by a pancreatic neuroendocrine tumor, progressing after a first line of therapy with cisplatin plus etoposide, achieving a clinical and radiological response with metronomic temozolomide “one-week-on/on-week-off regimen”, with continuing tumor shrinkage at 18 months from the beginning of the treatment. The intermittent dosing was chosen to reduce the frequency and severity of hematologic toxicity of TMZ as compared with more extended dosing schedules such as the 21/28-day or extended daily schedules, and for the poor Performance Status (PS = 2) of the patient at the beginning of the treatment.

According to the present knowledge it wouldn’t be rash to claim that the treatment with TMZ can develop mechanisms of induction of the immune response, especially with metronomic schedule.

## Review

### Case report

**Fig. 1:**
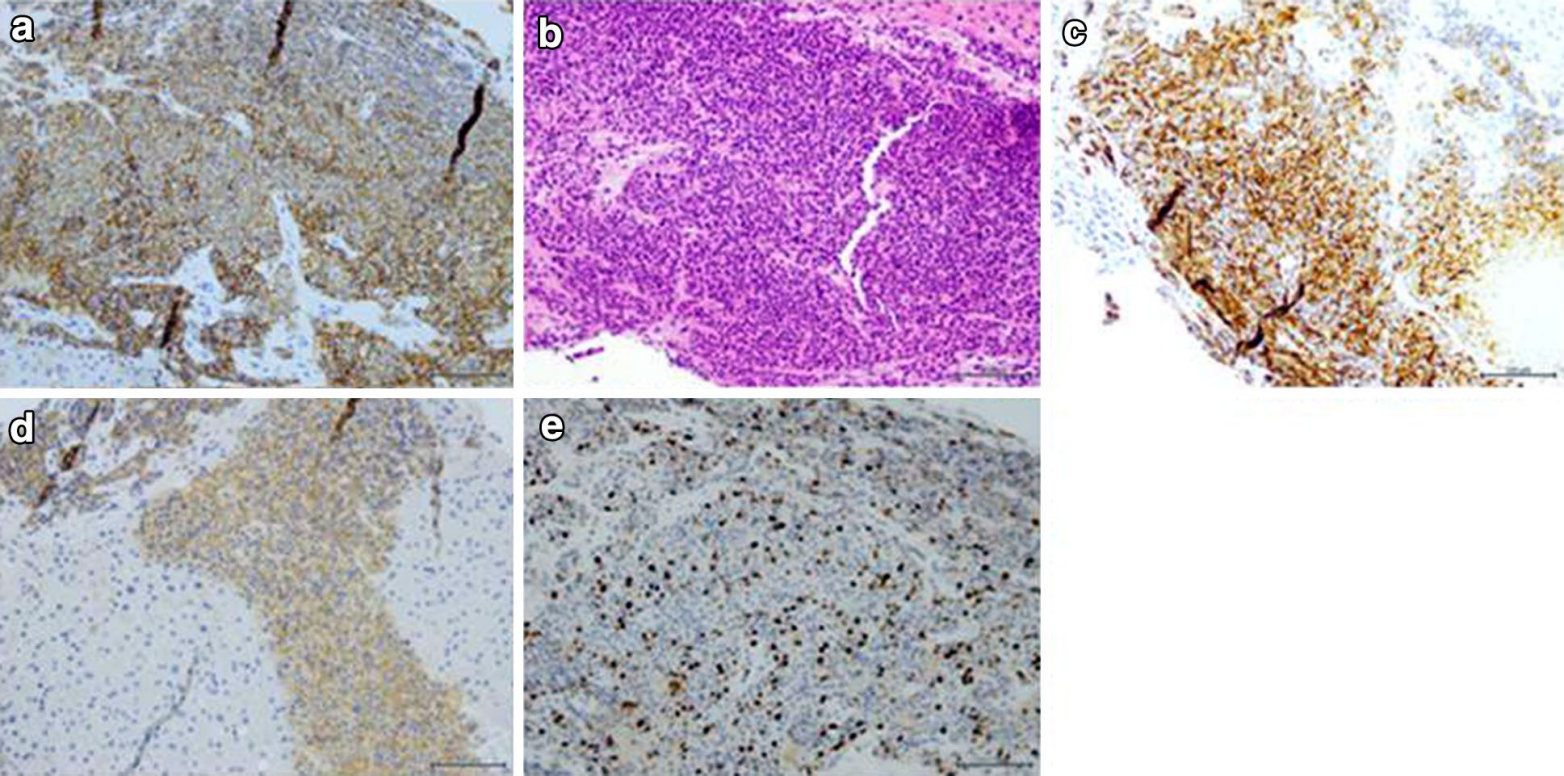
Histological examination with immunohistochemistry and hematoxylin-eosin staining (e/e). **a** CD 56 20 × magnification; **b** e/e 20 × magnification; **c** chromogranin 20 × magnification; **d** Synaptophysin 20 × magnification; **e** Ki-67 40 × magnification

**Fig. 2:**
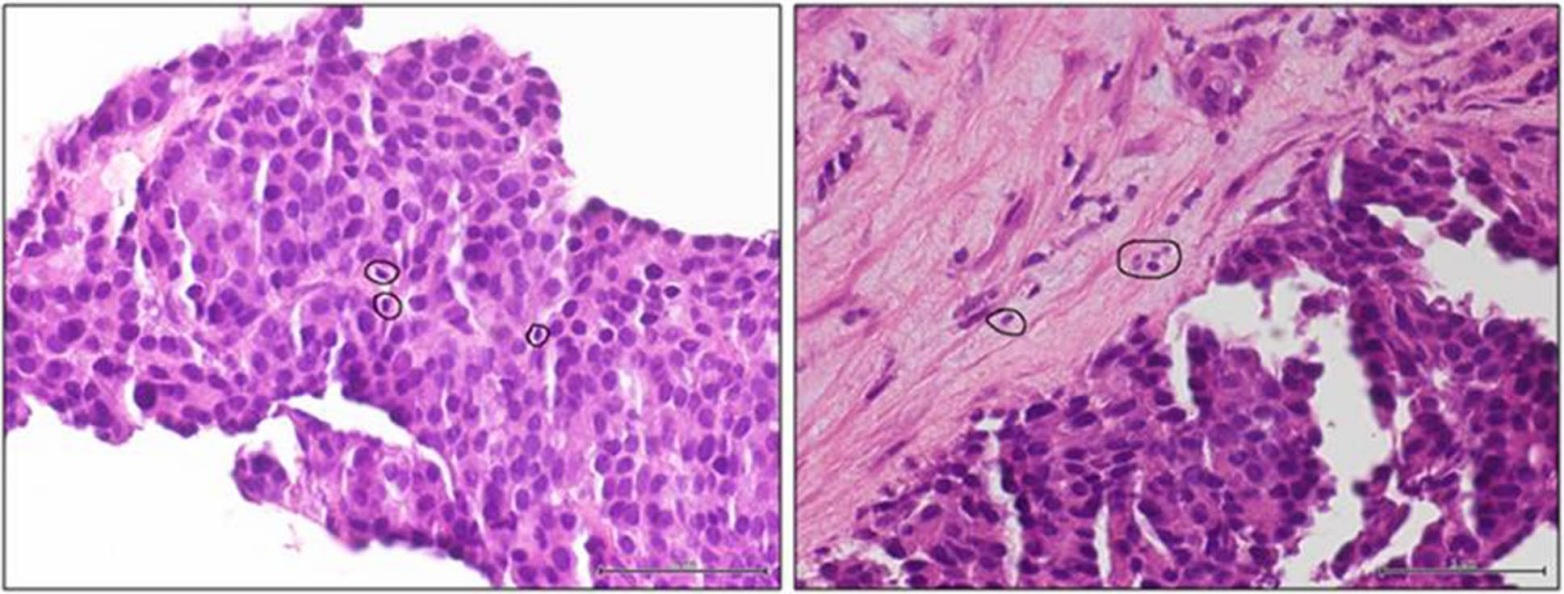
Peritumoral lymphocytes and leucocytes infiltrating tumor microenviroment. Tumor-infiltrating CD8 + T cells (*circles*) have been detected in the pNEC tumor biopsy of the patient. These cells infiltrating tumor micro environment are frequently associated with favorable clinical outcome in a remarkably large spectrum of cancers as MCC and pNEC

On September 2012 a 57-year-old female patient, due to repeated episodes of heartburn and dyspepsia, underwent endoscopy examination of the stomach that diagnosed the presence of gastritis with duodenal ulcer and Helicobacter Pilory (HP) infection. Blood tests showed an increase of AST, ALT and amylase. She also practiced an abdomen ultrasound that resulted negative for metastatic lesions. Therefore, was prescribed antibiotic therapy for HP eradication and introduced a regimen with protonic pump inhibitor (PPI). On July 2013, due to the worsening of nausea and vomiting was performed a total body *CT*-scan, which evidenced the presence of an advanced neoplasia of the pancreatic head with lymph nodal, liver, spleen and lung metastases. Hematology showed high levels of chromogranin A up to 371.0 U/L (n.v. 2.0 to 18.0) and 5HIAA up to 18 (<8). Then a liver biopsy under ultrasound guidance was performed. The histological examination diagnosed the presence of a poorly differentiated, endocrine-small cell carcinoma, (NEC) (CD56+; chromogranin +, synaptophysin +, CK7−; Ki67 >20 %) (Fig. 1) and also detected peritumoral lymphocytes and leukocytes infiltrating tumor micro environment (Fig. 2).

An OctreoScan performed on July 18 2013 showed the presence of hypermetabolic indicator receptor on the right subclavian region, retrotracheal, subcarinal, epi-mesogastric region (pancreas and lymphnodes), on multiple areas of the liver and on some skeletal sites (skull, dorsal vertebra and pelvis).

A PET-FDG practiced on July 20, 2013 showed pathological uptake at the pancreas (SUV max 4), at some liver lesions (SUV max 3) and at multiple skeletal localizations, in particular on the right humerus, on D4 and on the left acetabulum.

On July 27 2013, the patient arrived at Our Institute presenting highly symptomatic disease (vomiting G2, diarrhea G2, heartburn); laboratory tests showed elevation of AST (=93), ALT (=156) and GGT (=321). Therefore on July 20 2013 was prescribed treatment with octreotide LAR 30 every 4 weeks (q28) and on July 31 2013 the patient started a first-line chemotherapy with the combination cisplatin plus etoposide.

On March 2014 after six cycles of chemotherapy, the patient underwent total body *CT*-scan that showed radiological stable disease, with clinical response for the resolution of the disease-related symptoms and normalization of liver function tests. The patient continued the treatment with octreotide LAR 30 q28 but on June 2014, after three months of therapy there was a clinical progression of disease with worsening of performance status (ECOG PS = 2).

**Fig. 3:**
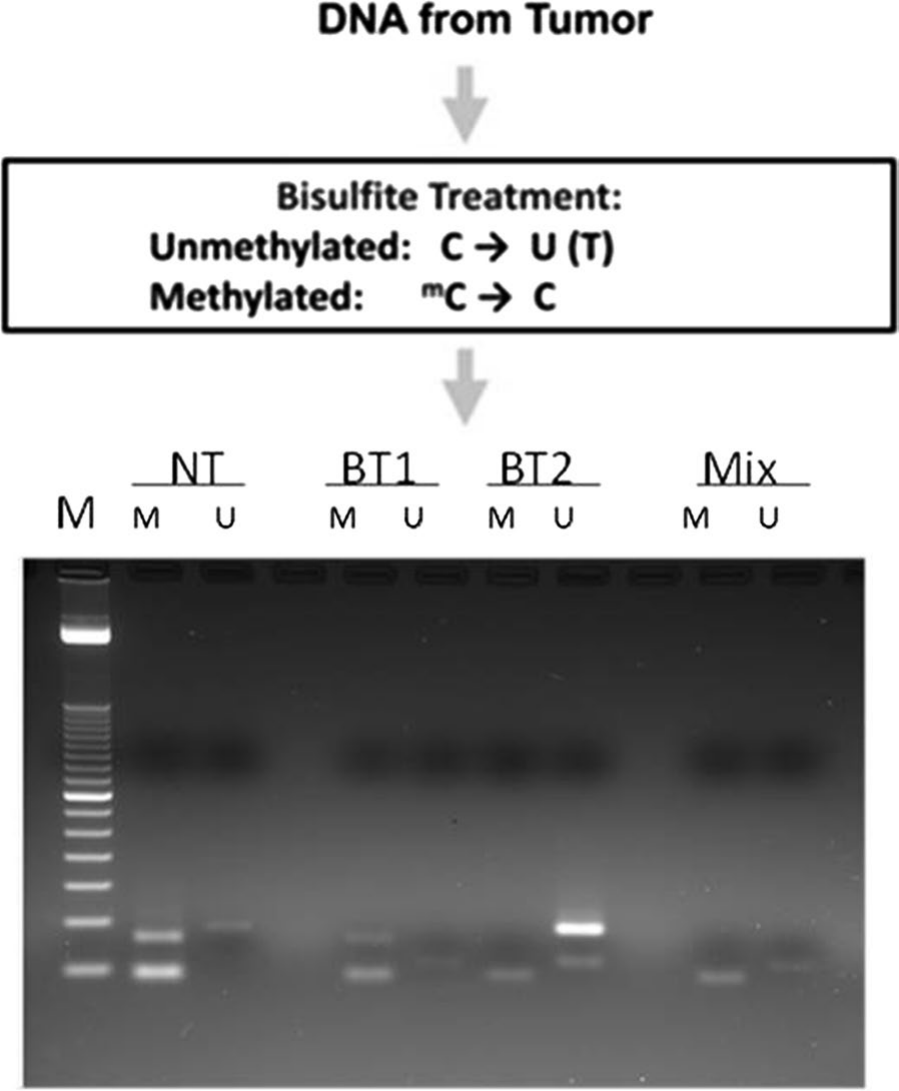
Detection of MGMT methylation by MSP and MethyLight qMSP. Isolated DNA from tumor specimen is treated with sodium bisulfite, which changes unmethylated cytosine (C) into uracil (U), but not methylated cytosine (^m^C), which remains unchanged. The modified DNA is used as a template for MSP or MethyLight qMSP. (MSP/agarose gel electrophoresis) When MGMT in neuroendocrine tumor is methylated (DNA M), the band is high, while the unmethylated band (DNA U) is low. The gel shows the presence of methylation band in BT1, brain tumor a with strong methylated, DNA M, used as positive control, and unmethylation band in BT2, brain tumor a with strong unmethylated, DNA U, used as negative control

If the first line of chemotherapy in neuroendocrine carcinomas with cisplatin and etoposide is not a real standard therapy, a second line therapy for NEC progressing patients does not really exist. Thus, considering the poor clinical status, we prescribed a treatment with temozolomide 75 mg/m2 “one week on/one week off”. On July 2014 the patient started the treatment. Metronomic temozolomide was well tolerated and no drug-related side effects were reported by the patient. The detection of MGMT methylation, by MSP and MethyLight qMSP, resulted positive (Fig. 3).

**Fig. 4:**
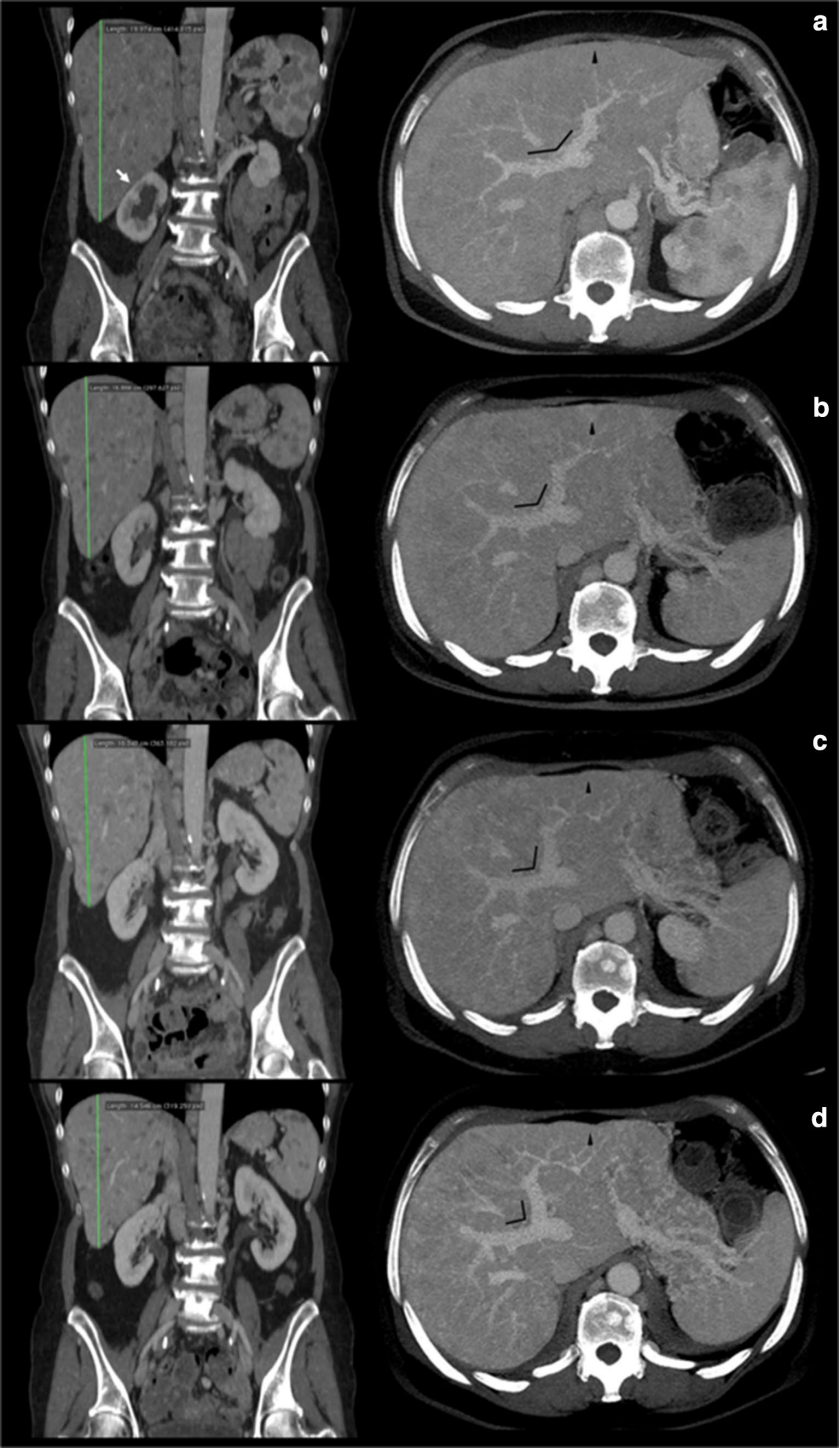
Hepatomegaly reduction. CT portal-phase contrast-enhanced images: Coronal plane reconstruction and Axial Maximum Intensity Projection (MIP) reconstruction at time 0 (**a**) and at 4 (**b**), 7 (**c**), 10 month (**d**) follow up examinations. Progressive reduction of longitudinal diameter of the liver measured in the mid-clavicular line was observed at follow up study. Progressive reduction of lower margin extension below costal arch was also detected. Caudal displacement of right kidney at time 0 (*white arrow*) gradually disappears at successive studies. Axial MIP images show progressive reduction of the displacement effect on the portal bifurcation due to decrease of metastatic parenchymal lesions: space between left and right portal trunk (*angle*) gradually decrease over time. Note the increased caliber of portal trunks and progressive reduction of the swelling of liver profile (*arrowhead*) related with hepatomegaly, with increased thickness of perivisceral fat

**Fig. 5:**
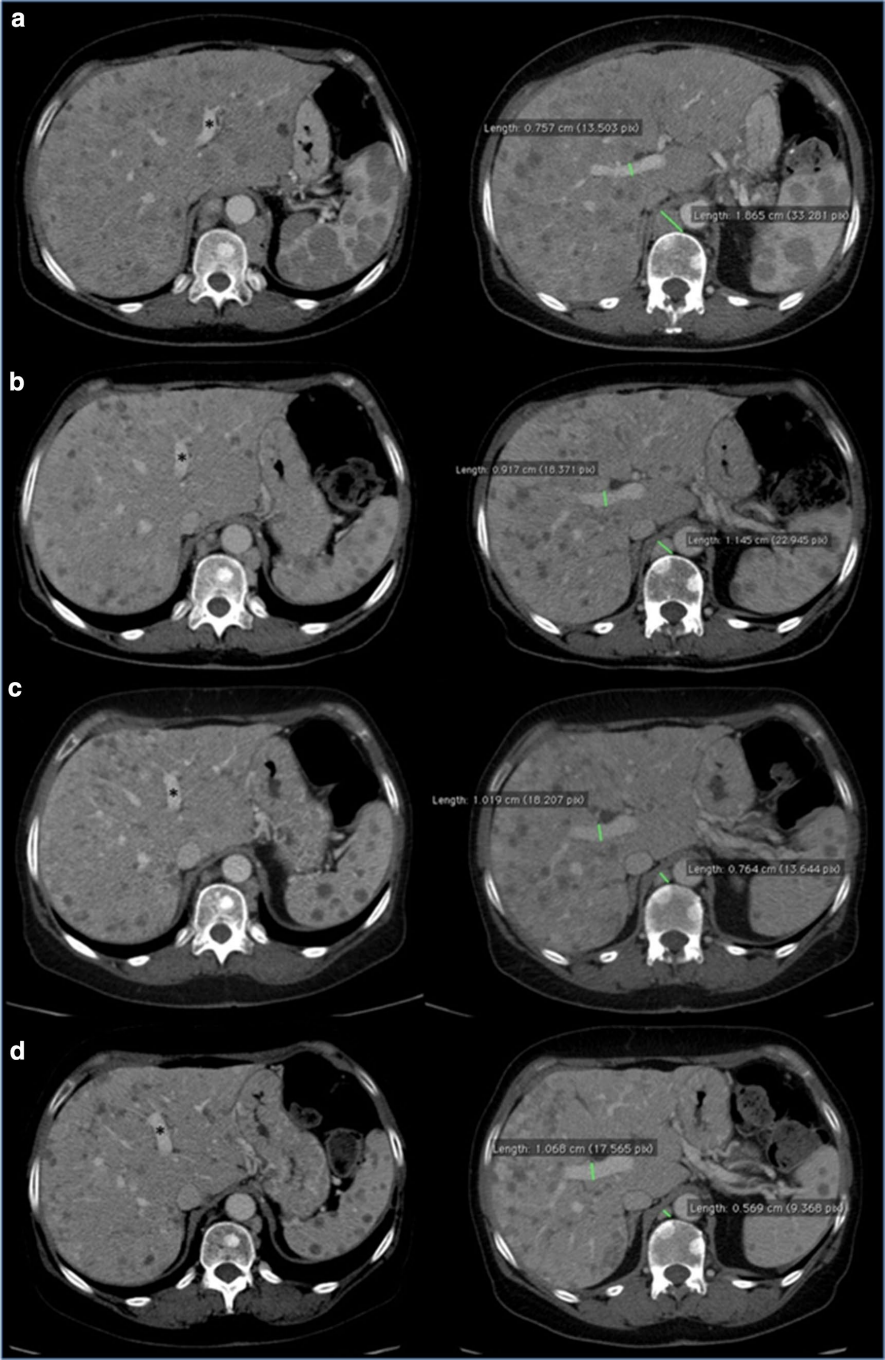
CT portal-phase contrast-enhanced images of two different planes at time 0 (**a**) and at 4 (**b**), 7 (**c**), 10 month (**d**) follow up examinations. Conspicuous volume reduction of paraesophageal and paracardial lymph nodes was observed over time. Increased caliber of portal trunks was detected, due to parenchymal architecture changes, reduced mass effect and mild portal hypertension. Note progressive dislocation of left portal trunk *asterisk* from *left* to *right* side due to reduction of hepatomegaly. Also note volume decrease of liver and spleen lesions with gradually remission of parenchymal architecture

**Fig. 6:**
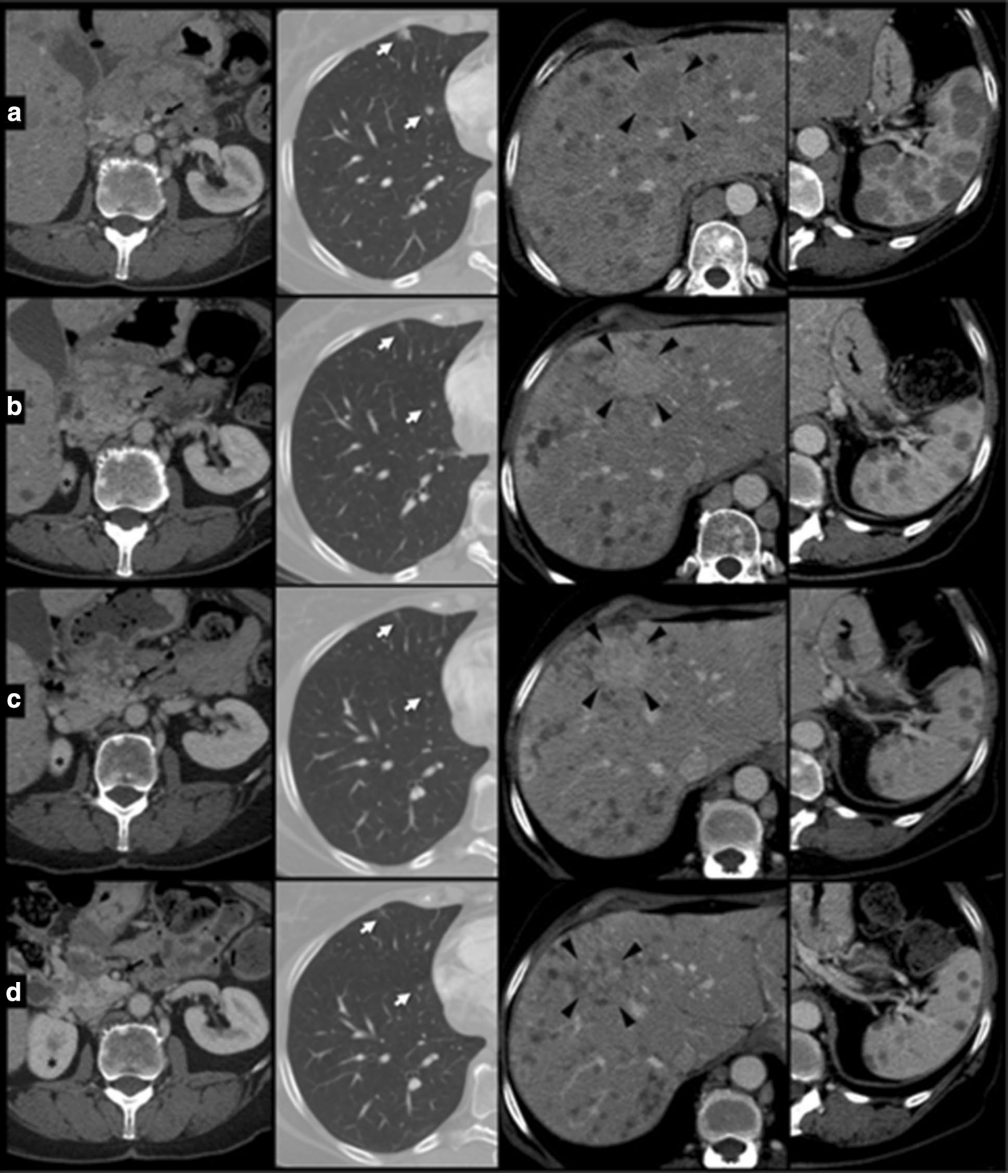
Changes of lesions over time in pancreatic head region, lung, liver and spleen at time 0 (**a**) and at 4 (**b**), 7 (**c**), 10 month (**d**) follow up examinations. Pancreas: enlargement of pancreatic head region was observed at time 0, with strongly inhomogeneous density, indistinct pancreatic margins and surrounding retroperitoneal fat stranding; some peripancreatic lymph nodes enlarged were detected. Following CT examinations show gradually decrease of pancreatic swelling with better definition of parenchymal lesions, going towards progressive regression e colliquation. Note progressive appearance of right kidney’s *upper pole asterisk*, according to reduction of displacement effect by liver. Furthermore, note the displacement of superior mesenteric artery (*black arrow*) to right side due to reduction of pancreatic swelling. Lung: progressive volume reduction of metastatic lesions (*white arrow*) in anterior basal segment of right lower pulmonary lobe. Liver: one of the major lesions of the liver at the IV segment (*arrowhead*) underwent progressive volume reducing ad enhancement pattern. Spleen: volume reduction of multiple metastatic lesions

On August 2014, the patient had a significant clinical benefit with improvement of performance status (ECOG PS: 0). The total body *CT*-scan performed on October 2014 evidenced a RECIST partial response (Figs. 4, 5, 6) with reduction in number and volume of liver and spleen metastases, reduction of hepatomegaly and splenomegaly, reduction of periesofageal, retrocrural and lomboaortic lymphnodal metastases, reduction of lung metastases, disappearance of ascites, significantly reduction of the lesion of the pancreatic head.

The patient continued the treatment and the total body *CT*-scan performed on January 2015 showed once again the persistence of the framework of further instrumental disease response, on lung, liver, and multiple lymphnodal metastases. Then, given the response both clinical and radiological and considering the improvement in quality of life and Performance Status achieved by the patient, we continued the treatment with temozolomide at the same dosage and regimen. In April 2015, the patient, stable for clinical conditions (PS = 0), with very good quality of life, performed a new total body *CT*-scan (Figs. 4, 5, 6) which showed an unexpected, further partial response. Treatment with TMZ is still ongoing.

## Discussion

Despite new promising therapeutic strategies in the setting of G1 – G2 pNET, recurrent or metastatic pancreatic Neuroendocrine Carcinoma (pNEC) continues to be an incurable disease with poor prognosis and there is no standard option for second line chemotherapy. Ongoing clinical trials are testing the efficacy of immune modulating antibodies against the PD-1/PDL-1 pathway (i.e. Avelumab in Merkel Cell Carcinoma) in pre-treated, progressing neuroendocrine tumors (NCT01772004, NCT01375842).

Few prospective studies that investigate a second line chemotherapy in neuroendocrine tumors are available.

In 2011 Welin et al. [25] showed that Temozolomide may be an active, well tolerated, second-line chemotherapy regimen for NEC patients (mainly gastrointestinal) who have progressed after first-line chemotherapy. It seems to be effective also in lung carcinoid.

Early clinical studies [26, 27] investigating shortened and extended dosing schedules suggested that continuous daily administration of temozolomide resulted more effective than a single dose. More frequent administration (e.g., twice a daily) yielded higher levels of O6-methylguanine DNA adducts, suggesting that the capacity of tumor cells to repair these adducts can be saturated. Unfortunately, hematologic toxicity was dose limiting of some schedules.

In Glioma, it has been suggested that intermittent dosing (one week on/one week off treatment) may reduce the frequency and severity of hematologic toxicity compared with more extended dosing schedules such as the 21/28-day or extended daily schedules [28].

Several studies [28] have shown that prolonged exposure to temozolomide can deplete MGMT activity in blood cells, a process that could potentially increase the antitumor activity of the drug. To date, however, there are limited data demonstrating the depletion of MGMT activity in tumor tissue exposed to temozolomide. Wolfgang [28] et al. studied in patients with glioma the effects of the treatment with either an alternating weekly schedule (7 days on/7 days off) or for 21 consecutive days every 28-day cycle (21/28-day schedule) on MGMT enzyme activity assayed in peripheral blood mononuclear cells (PBMCs). The results showed a time- and dose-dependent decrease in MGMT activity with both regimens.

A variety of dosing schedules that increase the duration of exposure and the cumulative dose of temozolomide are currently being investigated for the treatment of glioma, with the goal of improving antitumor activity and overcoming resistance [29–32]. These alternative dosing regimens have been shown to deplete MGMT activity in peripheral blood mononuclear cells, but the regimen that provides the best balance between enhanced antitumor activity and acceptable hematologic toxicity has yet to be determined.

According with these considerations, we decided to use the regimen “Seven-Days-On/Seven-Days-Off Regimen” because it appears to reduce the frequency and severity of hematologic toxicity as compared with more extended dosing schedules such as the 21/28-day or extended daily schedules.

In our case, we treated a metastatic pNEC patient, progressing after a first line platinum-based chemotherapy, with metronomic regimen (75 mg/m^2^/day) of temozolomide “one week on/one week off”.

After 1 month of treatment, a clinical response, with regression of disease-related symptoms and performance status improvement from ECOG PS 2 to 0 was obtained. After 3 months of therapy a RECIST partial response was observed. The treatment was well tolerated without drug-related side effects. After 18 months of therapy the partial response goes on.

Though on the tumor samples of patient the detection test of MGMT methylation was positive, we think that this result cannot be due only to the action of the alkylating drug, but needs immunological implications of the chemotherapy.

Conventional anticancer chemotherapy is generally thought to act through selective killing of tumor cells or by irreversibly arresting their growth. Cytotoxic drugs act in different phases of cell cycle interfering with DNA synthesis, or inducing a damage on DNA, leading to tumor cell death. Always more evidences indicate that several chemotherapeutic agents are more active against tumors implanted in immunocompetent hosts as compared with tumors in immunodeficient hosts. This clearly indicates the existence of a correlation between the activity of chemotherapeutic agents and the hosts’ immune system [28].

Pilot clinical trials with cancer vaccines gave clear evidence of the positive impact of chemotherapy on antitumor immune responses. Gene expression analysis of peripheral blood mononuclear cells (PBMCs) from melanoma patients treated with dacarbazine and a peptide-based vaccine revealed, by one day after chemotherapy, increased expression of immunoregulatory factors that can account for the enhancement of tumor antigen-specific CD8 T cell responses observed in those patients, as compared with patients treated with vaccine alone [33].

Together with these effects were resulted a widening of the antigenic repertoire and an expansion of antigen-specific T-cell tumor reactivity [34].

Moreover cancer often results in an imbalance of Th1/Th2 immunity, which can be restored by some antineoplastic drugs. Besides the active stimulation of effector cells, immune-potentiation by cytotoxic chemotherapy can also be achieved through the inhibition of tumor-induced immune suppression.

Several subsets of immunoregulatory cells have been identified so far in cancer patients [35].

CD4-CD25-expressing Tregs and myeloid cells with suppressive functions, namely myeloid-derived suppressive cells (MDSCs) and tumor-associated macrophages (TAMs), accumulate in the blood and, especially, within tumor burden, thus contributing to disease progression through various mechanisms. Gemcitabine kills MDSCs, both in vitro and in vivo [36–38] with no significant reduction in other cell subsets. The selective loss of MDSCs was accompanied by an increase in the antitumor activity of CD8 T and NK cells.

It has been demonstrated that metronomic temozolomide, reduces the number and the suppressive function of circulating Tregs in rats bearing glioma, although it did not restrain tumor growth [39].

Under defined circumstances, chemotherapy-induced tumor cell death can set the stage for an effective antitumor immune response. In fact some chemotherapeutics, including anthracyclines, oxaliplatin and CTX, are unique in their capacity to induce an immunogenic type of tumor cell death [40, 41] thereby converting dying tumor cells into adjuvanted-endogenous vaccines.

An interesting thing to mention is that, the count of lymphocytes (ALC) and monocytes (AML) in blood samples of our patient has been evaluated, during these months. There has been an initial increase and a subsequent stabilization of these values over time (Table 2), and this is in line with the above hypothesis.

**Table 2:** Values of white blood cells, lymphocytes and monocytes

Date	WBC	ALC	AML
07/15/2014	5910	1040 (17.7 %)	367 (6.21 %)
8/05/2014	11,500	1070 (9.3 %)	878 (7.63 %)
12/11/2014	6950	1459 (21.0 %)	688 (9.9 %)
02/16/2015	5760	826 (14.3 %)	487 (8.44 %)
04/17/2015	6000	756 (14.6 %)	489 (8.15 %)
06/12/2015	4670	655 (14 %)	453 (9.63 %)
07/28/2015	5170	676 (13.1)	435 (8.41)
11/17/2015	6560	800 (11.4 %)	525 (8.0 %)
01/12/2016	6990	997 (14.3)	603 (8.63 %)

The table shows an increase of the percentage values of lymphocytes (ALC) and monocytes (AML) in patient blood sample, and a subsequent stabilization of these values over time

*WBC* white blood cells; *ALC* absolute limphocyte count; *AMC* absolute monocyte count

Chemotherapy agents have a significant impact on both tumor and host immune system. Even if no systematic analysis has been performed to evaluate differences in the immune-based effects of conventional chemotherapeutic agents depending on cancer histology or stage, it is now clear that the existence of tumor–host interplay influences the magnitude, quality and efficacy of most anticancer strategies. Advances in tumor immunology have now explained some key mechanisms that represent the basis of therapeutic synergy with other treatments.

In our clinical case, the continuous response after 18 months of treatment, associated with the clinical benefit obtained, indicate a plausible immune activation induced by metronomic temozolomide. Moreover this case report highlights the efficacy and tolerability of this regimen even in a patient with poor performance status and in this particular category of neoplasms, opening new scenarios of treatment for metastatic pNET.

Therefore, this regimen has a promising activity that should be evaluated in further studies to confirm the efficacy and safety of temozolomide as second-line treatment of Gastro-entero-Pancreatic Neuroendocrine Carcinomas progressing after first-line Platinum-based therapy, especially in selected patients, such as those who have levels of MGMT methylation. A phase II clinical trial using temozolomide as second line of NEC progressing after platinum-based first line chemotherapy, has been designed (TENEC trial).

## Conclusion

This case report highlights the efficacy and tolerability of metronomic temozolomide even in this particular category of neoplasms and in this therapeutic setting, opening new scenarios of treatment of metastatic pNET.

Therefore, this regimen has a promising activity that should be evaluated in further studies to confirm the efficacy and safety of Temozolomide for second-line treatment of Gastro-entero-Pancreatic Neuroendocrine Carcinomas progressing after first-line Platinum-based therapy, especially in selected patients, such as those who have levels of MGMT methylation.

Recently, a phase II study TENEC, temozolomide as second line of NEC in progression after platinum-based first line, at our Institution was started.

## Appendix: Methods

DNA extraction and bisulfite treatment. To ensure high tumour DNA content, FFPE tissue sections were stained with H&E and histologically examined by an expert neuropathologist Sections showing a tumour cell content of more than 80 % were directly subjected to DNA extraction.. Extraction of genomic DNA was performed using the QIAamp DNA Mini Kit (Qiagen, Hilden, Germany) and quantified with a NanoDrop ND-1000 (PeqLab, Erlangen, Germany). One micrograms of extracted DNA as well as CpGenome Universal Methylated DNA (Chemicon International, Temecula, CA) and CpGenome Universal Unmethylated DNA (Chemicon International) as controls were subjected to bisulfite treatment using the EpiTect Bisulfite Kit (Qiagen). The bisulfite-treated DNA was used for Methylation-specific polymerase chain reaction (MSP//PCR). The two primer sets established by Esteller et al. for MSP of *MGMT* [2] were 5′-TTTCGACGTTCGTAGGTTTTCGC-3′ (forward primer) and 5′-GCACTCTTCCGAAAACGAAACG-3′ (reverse primer) for methylated template detection (M primers, product length 81 bp; and 5′-TTTGTGTTTTGATGTTTGTAGGTTTTTGT-3′ (forward primer) and 5′-AACTCCACACTCTTCCAAAAACAAAACA-3′ (reverse primer) for unmethylated template detection (U primers, product length 93 bp;. The PCR was performed in a total volume of 20 µl containing 10 µl HotStarTaq Mix (Qiagen), 1 µl of the respective forward and reverse primer (10 mol), 6 µl high purity water and 2 µl bisulfite-treated template DNA. The PCR programme was 95 °C for 15 min, then 35 cycles of 95 °C for 50 s, 59 °C for 50 s and 72 °C for 50 s, followed by a final step at 72 °C for 10 min. PCR reactions with CpGenome Universal Methylated DNA (Chemicon International), with CpGenome Universal Unmethylated DNA Vial A (Chemicon International), and without any DNA (non-template control) were included as controls. PCR products were separated on a 2 % agarose gel.

## Abbreviations

GEP: gastro-entero-pancreatic
SSA: somatostatin analog
TTP: time to progression
NET: neuroendocrine tumor
PRRT: peptide radionuclide receptor therapy
TMZ: temozolomide
ORR: overall response rate
AST: aspartato-transferasi
ALT: alanina-transferasi
PPI: proton pump inhibitor
5HIAA: hydroxyindoleacetic acid
GGT: gamma glutamyl transferase
NEC: neuroendocrine carcinoma
MGMT: methylguanine-DNA methyltransferase
PS: performance status
PBMCs: peripheral blood mononuclear cells
CTX: cyclophosphamide

## References

[1] Williams ED, Sandler M (1963). The classification of carcinoid tum ours. Lancet.

[2] Yao JC, Hassan M, Phan A (2008). One hundred years after“carcinoid”: epidemiology of and prognostic factors for neuroendocrine tumors in 35,825 cases in the United States. J Clin Oncol.

[3] Gastrointestinal Pathology Study Group of Korean Society of Pathologists, Cho MY, Kim JM, et al (2012). Current trends of the incidence and pathological diagnosis of gastroenteropancreatic neuroendocrine tumors (GEPNETs)in Korea 2000–2009: multicenter study. Cancer Res Treat.

[4] Bernick PE, Klimstra DS, Shia J (2004). Neuroendocrine carcinomas of the colon and rectum. Dis Colon Rectum.

[5] Milan SA, Yeo CJ (2012). Neuroendocrine tumors of the pancreas. Curr Opin Oncol.

[6] Yao JC, Eisner MP, Leary C (2007). Population-based study of islet cell carcinoma. Ann Surg Oncol.

[7] Rinke A, Müller H-H, Schade-Brittinger C (2009). Placebo-controlled, double-blind, prospective, randomized study on the effect of octreotide LAR in the control of tumor growth in patients with metastatic neuroendocrine midgut tumors: a report from the PROMID study group. JCO.

[8] Caplin ME, Pavel M, Ćwikła JB (2014). Lanreotide in metastatic enteropancreatic neuroendocrine tumors. N Engl J Med..

[9] Toumpanakis (2007). Cytotoxic treatment including embolization/chemoembolization for neuroendocrine tumours. Best Pract Res Clin Endocrinol Metab..

[10] Nisa (2011). Yttrium-90 DOTATOC therapy in GEP-NET and other SST2 expressing tumors: a selected review. Ann Nucl Med.

[11] Moertel CG, Kvols LK, O’Connell MJ (1991). Treatment of neuro-endocrine carcinomas with combined etoposide and cisplatin. Evidence of major therapeutic activity in the anaplastic variants of these neoplasms. Abstr US Endocr Soc.

[12] Ridolfi L, Petrini M, Granato AM (2013). Low-dose temozolomide before dendritic-cell vaccination reduces (specifically) CD4+CD25++Foxp3+ regulatory T-cells in advanced melanoma patients. J Transl Med.

[13] Kulke MH, Stuart K, Enzinger PC, Ryan DP, Clark JW, Muzikansky A (2006). Phase II study of temozolomide and thalidomide in patients with metastatic neuroendocrine tumors. J Clin Oncol.

[14] Ekeblad S, Sundin A, Janson ET, Welin S, Granberg D, Kindmark H (2007). Temozolomide as monotherapy is effective in treatment of advanced malignant neuroendocrine tumors. Clin Cancer Res.

[15] Kulke M, Blaszkowsky LS, Zhu AX, et al. Phase I/II study of everolimus (RAD001) in combination with temozolomide (TMZ) in patients (pts) with advanced pancreatic neuroendocrine tumors (NET). 2010 ASCO Gastrointestinal Cancers Symposium, January 22-24, 2010 (Abstract).

[16] Koumarianou A, Antoniou S, Kanakis G, Economopoulos N, Rontogianni D, Ntavatzikos A (2012). Combination treatment with metronomic temozolomide, bevacizumab and long-acting octreotide for malignant neuroendocrine tumours. Endocr Relat Cancer.

[17] Strosberg JR, Fine RL, Choi J, Nasir A, Coppola D, Chen DT (2011). First-line chemotherapy with capecitabine and temozolomide in patients with metastatic pancreatic endocrine carcinomas. Abstr US Endocr Soc.

[18] Welin S, Sorbye H, Sebjornsen S, Knappskog S, Busch C, Oberg K (2011). Clinical effect of temozolomide-based chemotherapy in poorly differentiated endocrine carcinoma after progression on first-line chemotherapy. Abstr US Endocr Soc.

[19] Gounaris I, Rahamim J, Shivasankar S, Earl S, Lyons B, Yiannakis D (2007). Marked response to a cisplatin/docetaxel/temozolomide combination in a heavily pretreated patient with metastatic large cell neuroendocrine lung carcinoma. Anticancer Drugs.

[20] Bravo EL, Kalmadi SR, Gill I (2009). Clinical utility of temozolomide in the treatment of malignant paraganglioma: a preliminary report. Horm Metab Res.

[21] Lindholm DP, Eriksson B, Granberg D (2012). Response to temozolomide and bevacizumab in a patient with poorly differentiated neuroendocrine carcinoma. Med Oncol.

[22] Isacof WH, Moss RA, Pecora AL, Fine L. Temozolomide/capcitabine therapy for metastatic neuroendocrine tumors of the pancreas: a retrospective review. J Clin Oncol. 2006;24(20 Suppl):Abstr 14023.

[23] Strosberg JR, Fine RL, Choi J, Nasir A, Coppola D, Chen DT, Helm J, Kvols L (2011). First-line chemotherapy with capecitabine and temozolomide in patients with metastatic pancreatic endocrine carcinomas. Cancer.

[24] Ott PA, Maria Elez-Fernandez ME, Hiret S, Kim DW, Moss RA, Winser T, et al. Pembrolizumab (MK-3475) in patients (pts) with extensive-stage small cell lung cancer (SCLC): Preliminary safety and efficacy results from KEYNOTE-028. J Clin Oncol. 2015;33:abstr 7502.

[25] Welin S, Sorbye H, Sebjornsen S, Knappskog S, Busch C, Oberg K (2011). Clinical effect of temozolomide-based chemotherapy in poorly differentiated endocrine carcinoma after progression on first-line chemotherapy. Abstr US Endocr Soc.

[26] Newlands ES, Blackledge GR, Slack JA (1992). Phase I trial of temozolomide(CCRG 81045: M&B 39831: NSC 362856). Br J Cancer.

[27] Newlands ES, Stevens MFG, Wedge SR, Wheelhouse RT, Brock C (1997). Temozolomide: a review of its discovery, chemical properties, preclinical development and clinical trials. Cancer Treat Rev.

[28] Wolfgang Wick, Michael Platten, and Michael Weller, New (alternative) temozolomide regimens for the treatment of glioma, Neuro-Oncology, 2009. 10.1215/15228517-2008-078 PMC2718961 18772354

[29] Caroli M, Locatelli M, Campanella R (2007). Temozolomide in glioblastoma: results of administration at first relapse and in newly diagnosed cases. Is still proposable an alternative schedule to concomitant protocol?. J Neurooncol.

[30] Chinot OL, Barrie M, Fuentes S (2007). Correlation between O6-methylguanine- DNA methyltransferase and survival in inoperable newly diagnosed glioblastoma patients treated with neoadjuvant temozolomide. J Clin Oncol.

[31] Combs SE, Gutwein S, Schulz-Ertner D (2005). Temozolomide combined with irradiation as postoperative treatment of primary glioblastoma multiforme. Phase I/II study. Strahlenther Onkol..

[32] Combs SE, Schulz-Ertner D, Welzel T, Bischof M, Debus J (2007). Re-irradiation using high precision radiotherapy and concomitant temozolomide in patients with recurrent glioma: re-challenge with radio-chemotherapy [abstract 12517]. J Clin Oncol.

[33] Tai-Gyu Kim,Chang-Hyun Kim,Jung-Sun Park,Sung-Dong Park,Chung Kwon Kim, Dong-Sup Chung,and Yong-Kil Hong (2010). Immunological Factors Relating to the Antitumor Effect of Temozolomide Chemoimmunotherapy in a Murine Glioma Model. Clin Vaccine Immunol.

[34] Nistico P, Capone I, Palermo B, Del Bello D, Ferraresi V, Moschella F (2009). Chemotherapy enhances vaccine-induced antitumor immunity in melanoma patients. Int J Cancer.

[35] Palermo B, Del Bello D, Sottini A, Serana F, Ghidini C, Gualtieri N (2010). Dacarbazine treatment before peptide vaccination enlarges T-cell repertoire diversity of melan-a- specific, tumor-reactive CTL in melanoma patients. Cancer Res.

[36] Poschke I, Mougiakakos D, Kiessling R (2011). Camouflage and sabotage: tumor escape from the immune system. Cancer Immunol Immunother.

[37] Vincent J, Mignot G, Chalmin F, Ladoire S, Bruchard M, Chevriaux A (2010). 5-Fluorouracil selectively kills tumor-associated myeloid-derived suppressor cells resulting in enhanced T-cell-dependent antitumor immunity. Cancer Res.

[38] Mundy-Bosse BL, Lesinski GB, Jaime-Ramirez AC, Benninger K, Khan M, Kuppusamy P (2011). Myeloid-derived suppressor cell inhibition of the IFN response in tumor-bearing mice. Cancer Res.

[39] Banissi C, Ghiringhelli F, Chen L, Carpentier AF (2009). Treg depletion with a low-dose metronomic temozolomide regimen in a rat glioma model. Cancer Immunol Immunother.

[40] Schiavoni G, Sistigu A, Valentini M, Mattei F, Sestili P, Spadaro F (2011). Cyclophosphamide synergizes with type I interferons through systemic dendritic cell reactivation and induction of immunogenic tumor apoptosis. Cancer Res.

[41] Galluzzi L, Senovilla L, Zitvogel L, Kroemer G (2012). The secret ally: immunostimulation by anticancer drugs. Nat Rev Drug Discov.

